# The increase in risk classification using Canada’s Guidance on Alcohol and Health: an empirical examination in a sample of community adults in Ontario

**DOI:** 10.24095/hpcdp.45.2.03

**Published:** 2025-02

**Authors:** Kyla L. Belisario, Amanda Doggett, James MacKillop

**Affiliations:** 1 Peter Boris Centre for Addictions Research, McMaster University; St. Joseph’s Healthcare Hamilton, Hamilton, Ontario, Canada; 2 Department of Psychiatry & Behavioural Neurosciences, McMaster University, Hamilton, Ontario, Canada

**Keywords:** drinking guidelines, alcohol consumption, patterns of alcohol use, risk perception

## Abstract

**Introduction:**

The 2023 Canadian Centre on Substance Use and Addiction drinking guidelines specify a universal low-risk threshold of 2 or fewer drinks per week, lower than previous guidelines that recommended no more than 10 drinks per week or 2 per occasion for females, and 15 per week or 3 per occasion for males. This study examined the increases in risk classification and perceptions of these new guideline thresholds.

**Methods:**

Prevalence of those exceeding the new low-risk threshold was compared with that of previous and other international guidelines in an observational cohort of community adults (N=1502) from southern Ontario who had been followed since 2018 (11 waves of data collection). To examine awareness of the new guidelines and perceived risk of drinking beyond them, a follow-up was conducted with a subset of the cohort, three months after the release of the guidelines (April 2023).

**Results:**

Across waves, on average, 52% exceeded the new low-risk threshold compared to 11% who exceeded previous guidelines. Other international guidelines classified, on average, 16% (US), 20% (UK) and 29% (WHO) of the sample as exceeding low-risk guidelines. Approximately half of study participants (51%) were aware of Canada’s new guidelines, but 77% perceived exceeding 2 drinks per week as having little to no risk.

**Conclusion:**

Over four times more adults exceeded the new low-risk drinking threshold compared to that of the previous Canadian guidelines. Additionally, more were classified as exceeding the new low-risk threshold compared to other international drinking thresholds. These results, combined with low perceptions of risk associated with consuming more than2 drinks per week, suggest that many Canadians are at risk of exceeding the new guidelines.

**Highlights**

- Using the new low-risk drinking definition outlined in the recent Canada’s Guidance on Alcohol and Health report, the proportion of individuals surpassing the 2-drink threshold in a sample of N = 1502 was more than a four-fold increase compared to the previous Canadian guidelines.
- Rates of drinking beyond the new guidelines in this sample were unequally distributed across sex and age, with males and adults aged 50 and older exceeding the guidelines at a higher rate compared to their counterparts.
- In a subsample of n = 1278, more than three-quarters perceived that exceeding the new 2-drink threshold had little to no risk, suggesting a need for greater public education surrounding alcohol-related harms, particularly among those who are more likely to exceed the new guidelines.

## Introduction

New guidelines* on alcohol consumption were released in January 2023^1^ by the Canadian Centre on Substance Use and Addiction (CCSA), providing an update to the previous drinking guidelines (DGs) from 2011. The plan to update the DGs was a collaborative effort between the CCSA, Health Canada and the Public Health Agency of Canada (PHAC) as a result of new data highlighting the risks associated with alcohol consumption,1 although Health Canada has yet to adopt these new guidelines formally.^2^,^3^

With over three-quarters of Canadians consuming alcohol at least annually,^4^ the new guidelines pertain to a large proportion of the population. The aims of DGs are to educate Canadians on the risks associated with alcohol consumption by providing guidance on levels of consumption that may pose acute or chronic risks for individual health. According to the CCSA’s final report, updates to the DGs are based on evidence surrounding the health impacts of alcohol, but with a focus on individual risk for morbidity and mortality.^1^ The changes reflect a shift away from the notion that health benefits may possibly be associated with modest alcohol consumption, and instead emphasize recent evidence that all alcohol consumption carries risk,^5^,^6^ especially for some population groups such as young adults.^7^

***Defining drinking guidelines***

Typically, DGs either define a ceiling for low-risk drinking (sometimes referred to as “drinking in moderation”) or lay out a continuum of risk. The former definition utilizes a single quantitative threshold, which is used to categorize alcohol consumption either as within or in excess of that threshold (such as higher-risk drinking). The latter portrays the dose-response relationship between risks and drinking, distinguishing between low-, moderate- and high-risk drinking. Some guidelines, such as those from the World Health Organization (WHO), go further to distinguish between high- and very high-risk drinking.^8^

Guidelines also often consist of both per occasion and weekly consumption thresholds, the former pertaining to acute harms (e.g. falls, motor vehicle collisions, perpetration of violence) and the latter pertaining to chronic harms (e.g. cancer risk, liver disease risk). Evidence suggests that when used in tandem, this combination better predicts potential harm than guidelines that focus solely on one or the other.^9^ Typically, weekly guidelines focus on defining an average number of drinks consumed, and per occasion guidelines address patterns of consumption. Specifically, per occasion limits address heavy episodic drinking (HED; also called “binge drinking”), which is associated with acute risks,^10^ particularly for young adults.^11^

***Comparing previous and current Canadian guidelines***

Compared to Canada’s 2011 low-risk drinking definition, the updated weekly guidelines use a continuum of risk, akin to WHO guidelines on alcohol consumption, but with a lower threshold for defining low-risk. The low-risk threshold is defined as no more than 2 standard drinks (Canadian standard drink=13.45 g of pure ethanol alcohol) per week,^1^ whereas previous DGs defined the low-risk drinking threshold as no more than 10 standard drinks per week for females, or 15 for males.^12^ Additionally, a new threshold for moderate-risk drinking was added, defined as between 3 and 6 standard drinks per week.^1^ These low- and moderate-risk weekly drinking thresholds are based on a 1/1000 and 1/100 lifetime risk of mortality, respectively, and take into account new evidence of alcohol-related morbidity and mortality, published after the release of the 2011 DGs.^1^

The updated per occasion drinking threshold is also lower, defined as no more than 2 standard drinks per occasion.^1^ The previous Canadian guidelines defined per occasion limits of 2 drinks per occasion for females and 3 for males,^12^ allowing for multiple drinking days within these per occasion limits prior to the weekly maximum limits being exceeded. However, with the new low-risk definitions, only one “per occasion” limit of 2 drinks is permissible before the weekly limits are exceeded. In this regard, the new low-risk weekly and per occasion thresholds are identical, and do not distinguish between patterns of use.

Previous Canadian guidelines also highlighted a second set of per occasion limits labelled “special occasions,” which aligned with other widely used definitions of HED (i.e. limits exceeded with 4 or 5 standard drinks for females or males, respectively).^13^ While the new guidelines are universal, citing negligible differences between females and males at the low end of alcohol consumption,^1^ previous guidelines included sex-specific thresholds for females and males. As a result, the new low-risk drinking threshold represents a greater reduction in drinks for males (an 87% reduction of 13 drinks per week) compared to females (an 80% reduction of 8 drinks per week).

Beyond the previous Canadian guidelines, the new guidelines’ thresholds are also lower than the WHO’s continuum of risk, as well as other widely used international drinking definitions from countries with similar drinking climates as Canada,^14^ such as the National Institute on Alcohol Abuse and Alcoholism (NIAAA) in the US, and the National Health Service (NHS) in the UK. These international drinking definitions were also chosen as a comparison to the Canadian DGs in this study, as they provide a more heterogeneous range in type of guideline. Specifically, the NIAAA definition is used to define heavy alcohol use, with the per occasion threshold leveraging the definition of binge drinking; the NHS provides a comparison definition whereby weekly and per occasion guidelines are universal for both females and males; and the WHO drinking levels provide an opportunity to compare Canada’s new guidelines with another drinking risk continuum. A detailed overview of these international guidelines converted to Canadian standard drinks is provided in the Results section.

***Study aims***

Since the revised guidelines set a lower low-risk drinking threshold, a greater proportion of Canadians will inherently be categorized as exceeding the low-risk drinking threshold, but the magnitude of this change in proportion, and its distribution across sex and age, is not yet well understood. The aim of the current study was to quantify the increases in classification rates in an ongoing observational cohort study of Canadian adults. 

Specifically, this study had three aims: 

(1) to examine the average overall prevalence of those in excess of the new low- and moderate-risk DGs and compare it with both the previous Canadian DGs and drinking definitions from the NIAAA, NHS and WHO; 

(2) to examine differences in prevalence by sex assigned at birth, given the change from sex-specific to universal guidelines, as well as differences in prevalence by age group, given the established differences in drinking patterns across adulthood and the large reduction in per occasion drinking limit definitions; and

(3) to measure general awareness of the new DGs released in January 2023 and perception of risk associated with the low- and moderate-drinking thresholds including risk perception by sex assigned at birth and age group. Although not an inherently longitudinal question, calculating prevalence using a longitudinal dataset was considered beneficial to reduce the temporal specificity of findings and generate reliable estimates across a wide time window. This is particularly relevant as drinking behaviour in Canada is seasonal,^15^,^16^ and varied over the acute phase of the COVID-19 pandemic.^17^,^18^

*Notably, Canada’s previous guidelines (and their associated reports) were referred to as the “Low-Risk Alcohol Drinking Guidelines,” while the new guidelines are called “Guidance on Alcohol and Health.” Despite the shift in terminology, because a low-risk threshold was still included in the new guidance,1 both new and previous guidelines will be referred to as “low-risk drinking thresholds” for simplicity.

## Methods

***Ethics approval***

This study was approved by the Hamilton Integrated Research Ethics Board (Protocol #4699).

***Participants and measures***

Participants were members of an ongoing longitudinal cohort study of community adults (N=1502) from southern Ontario, first recruited from a research registry that was established between 2016 and 2018 as a one-time, in-person assessment. The registry recruited nonclinical individuals from the surrounding community via advertisements (both print and online, including social media platforms) to collect various health indicators. Previous reports provide detailed information about the cohort,^19^ but the broad eligibility criteria were: age 18 to 65 years at time of enrollment; interest in participating in health research studies; and no medical conditions that would preclude participation in future research studies. 

At the launch of the cohort in September 2018, participants were 59.7% female,† 27.3% non-White and approximately 35years of age (mean [M]: 34.58, standard deviation [SD]: 13.93), with a median yearly household income of CAD 60000 to 74999, and median education of some postsecondary education. There were 11 waves of online data collection prior to the release of the updated guidelines; waves occurred every 3 or 6 months‡ from 2018 to 2022, with high retention of the N=1502 across survey waves (retention across waves:M: 91.3%, SD: 3.86%). To address aims 1 and 2 of the study, the percent of participants exceeding drinking definition thresholds at a given wave was first calculated and then averaged across the 11 waves. A subsample of participants (n=1278) in the next follow-up wave of the study was assessed in April 2023 (3months after the public release of the new DGs), providing insight on public awareness and perception of the guidelines to address aim 3 of this study. An overview of sample characteristics is given in the Results section.

Typical consumption of standard alcohol servings for each day of the calendar week was collected via the Daily Drinking Questionnaire (DDQ^20^). By asking participants to recall the number of standard drinks they typically consumed on each of the seven days of the week during the past 6 months (3 months for survey waves administered quarterly), it could be determined whether weekly limits, as well as “combined” (meaning either weekly or per occasion) limits were exceeded. To be classified as exceeding the combined drinking threshold, individuals only needed to exceed the per occasion (based on their sex assigned at birth) or the weekly low-risk thresholds, but not necessarily both. Notably, many studies may not have data on alcohol consumption per occasion; for comparability and clarity purposes, the proportion of individuals exceeding the weekly limit in this study are the main focus in the Results section. However, it is acknowledged that the combined limits leverage more information, and as such, parallel proportions of those exceeding combined weekly and/or occasional limits are provided in the tables and figures.

To assess whether the subsample of participants (n=1278) was aware of the new guidelines, participants were asked to respond “Yes” or “No” to the question, “Are you aware of the new guidance about alcohol consumption as published in Canada’s Guidance on Alcohol and Health report?” Participants were also asked two questions about the perceived risk of exceeding the new drinking thresholds: “How much do people risk harming themselves physically and in other ways when they have more than [two drinks/six drinks] of an alcoholic beverage per week?” These questions, which pertained to the low- and moderate-risk thresholds, respectively, mirror questions used in the National Survey on Drug Use and Health (NSDUH);^21^ responses were “No risk,” “Slight risk,” “Moderate risk” and “Great risk.” 

***Canadian standard drinks equivalency***

In order to make direct comparisons across international guidelines, thresholds were translated into Canadian standard drinks, defined as 13.45 g of pure ethanol alcohol, or, as one beer or cider (12oz. or 341mL, at 5% alcohol); one glass of wine (5 oz. or 142 mL, at 12% alcohol); or one shot of distilled alcohol (1.5 oz. or 43 mL, at 40% alcohol).^1^

The heavy drinking definition by the NIAAA in the US (where 1 standard drink is equivalent to 1.04 standard Canadian drinks) outlines a weekly limit of 7 and 14standard drinks and a per occasion limit of 3 and 4 standard drinks for females and males, respectively.^13^,^22^,^23^ The NHS in the UK indicates a universal limit of 14 units of alcohol (1 unit is equivalent to 0.6 standard Canadian drinks) over a minimum of 3 days per week.^24^,^25^

The WHO utilizes a continuum of risk, expressing their DGs as the average number of drinks consumed across drinking days, with a low-risk drinking threshold defined as no more than 20 g and 40 g per drinking day, and a medium-risk drinking threshold defined as no more than 40 g and 60 g per drinking day, for females and males, respectively. The WHO also has per occasion thresholds defined as no more than 40 g for females and 60 g for males.^8^

For comparative purposes, in instances in which guidelines represent a fractionated Canadian standard drink, or when varying limits are defined (e.g. “most days” and “special occasions” definitions), lower limits rounded down (i.e. the more conservative limits) were used. Table 2 outlines the different DGs examined, converted into Canadian standard drinks.

**Table 2:** Drinking guideline thresholds converted into Canadian standard drinks and prevalence multipliers based on a sample of N = 1502 participants from southern Ontario across 11 data-collection waves, 2018 to 2022

Guideline (country, year)	Weekly threshold	Per occasion threshold	Mean (min–max) prevalence multipliers^a ^
a. Weekly	b. Combined
Low-risk
CCSA: Guidance on Alcohol and Health—low-risk (Canada, 2023)	Maximum 2 standard drinks per week	Maximum 2 standard drinks on a given day	—	—
CCSA: Canada’s Low-Risk Alcohol Drinking Guidelines (Canada, 2011)	Maximum 10 standard drinks a week for females, or 15 for men	Maximum 2 standard drinks for females, 3 for males most days Maximum 3 standard drinks for females, 4 for males for special occasions	4.6 (4.0–4.6)	2.1 (1.9–2.3)
NIAAA: Heavy Alcohol Use (US, 2009)	Maximum 7 standard drinks per week for females, or 14 for men	Maximum 3 standard drinks for females, 4 for men	3.2 (3.1–3.3)	2.5 (2.3–2.6)
NHS: Low-Risk Drinking Advice (UK, 2016)	Maximum 8.3 standard drinks	Weekly drinks are to be consumed across a minimum of 3 days (implied maximum of 3 drinks on any given occasion)	2.6 (2.5–2.8)	2.1 (2.0–2.3)
WHO: Low Risk Drinking Category (Global, 2000)	Maximum 1.5 standard drinks for females, 3.0 for males, each drinking day per week	Maximum 3.0 standard drinks for females, 4.5 or men	1.8 (1.6–2.0)	1.8 (1.6–1.9)
Moderate-risk
CCSA: Guidance on Alcohol & Health—Moderate-Risk (Canada, 2023)	Maximum 6 standard drinks per week	Maximum 2 drinks on a given day	—	—
WHO: Medium Risk Drinking Category (Global, 2000)	Maximum 3.0 standard drinks for females, 4.5 for males each drinking day per week	Maximum 3.0 standard drinks for females, 4.5 for men	2.8 (2.3–3.0)	2.2 (1.9–2.4)

**Abbreviations: **CCSA, Canadian Centre on Substance Use and Addiction; NIAAA, National Institute on Alcohol Abuse and Alcoholism; NHS, National Health Service; UK, United Kingdom, US, United States of America; WHO, World Health Organization. 

**Notes: **Drinking guideline (DG) definitions, alongside prevalence multipliers at which the guideline would exceed classifying those by either the (a) weekly drinking threshold; or (b) combined (either weekly or per occasion) drinking threshold using the new 2023 Canadian low-risk and moderate-risk drinking definitions. 

^a^ Prevalence multipliers can be interpreted as X number of times higher individuals in the sample would be classified as exceeding the new 2023 Canadian DGs relative to the comparison guidelines, and is calculated by dividing the average proportion of those exceeding the new 2023 drinking threshold by the average proportion of those exceeding the comparison guidelines. 

***Analyses***

For aims 1 and 2 of the study, the mean average proportion of participants exceeding guidelines at each wave was used to calculate the prevalence at which the sample exceeds the new guidelines relative to the previous and international guidelines. For aim 2, sex assigned at birth and age at the time of the assessment were used to classify participants into those aged under 30years, those aged 30 to 49 and those aged 50 or older. For aim 3, logistic regression was used to calculate the odds ratio of perceiving the different thresholds as risky by sex assigned at birth and age category (based on age at assessment), while controlling for reported alcohol use, awareness of the new guidelines, education level and household income as self-reported at the time of the assessment.

†The congruence between sex assigned at birth and cis-gender in this sample is high (99%). Since drinking definitions are generally based on biological factors rather than sociocultural differences, sex assigned at birth was chosen for analysis. However, this is not intended to diminish gender-specific risks, or the existence of other sexes outside of the binary of female and male (and genders outside of women and men).‡Each wave of data collection was scheduled to occur biannually; however, with the onset of the COVID-19 pandemic, two additional waves of data collection were added in July 2020 and January 2021, shortening the interval between adjacent assessments to 3 months.

## Results

***Overall drinking characteristics***

On average, 74% (range across 11 waves: 67%–80%) of the sample reported drinking at least one standard drink per week (see Supplemental Figure S1 at https://osf.io/57e94/?view_only=a8d2ed52c74b43b1b5262f59788c0c65). Although not recruited to be a representative national sample, the prevalence of adults endorsing alcohol use in the sample reflect Ontario provincial trends (74%) and national Canadian trends of use of between 76% and 78%, as estimated by the 2019 Canadian Alcohol and Drugs Survey (CADS).^4^ Among those in the sample who reported alcohol consumption, the mean average number of standard drinks consumed per week across all waves was 7.0 (mean average minimum and maximum across all waves: 6.5–7.9), and drinks were consumed across an average of 3.0 (mean average minimum and maximum across all waves: 2.8–3.4) days per week (Table 1). The highest reported per occasion consumption among those who consumed alcohol was, on average, 2.7 drinks (mean average minimum and maximum across all waves: 2.5–3.2).

**Table 1:** Demographics and mean summary statistics of drinking-related outcomes in a sample and subsample of community adults from southern Ontario, Canada

	Overall sample (N=1502)	Attitudes and perceptions subsample (n=1278)
Demographics	(Sept. 2018)	(Apr. 2023)
N (%) Female	896 (59.7)	786 (61.5)
N (%) Non-White	309 (21.6)	265 (20.7)
Median yearly household income (CAD)	60000–74999	90000–105000^a ^
Mean (SD) age	34.58 (13.93)	39.78 (14.14)
N (%) <30 years of age	761 (50.67)	466 (36.46)
N (%) 30–49 years of age	423 (28.16)	469 (36.70)
N (%) 50+ years of age	318 (21.17)	343 (26.84)
Drinking-related outcomes	Across waves (Sept. 2018–Oct. 2022)	(Apr. 2023)
Drinks per week, mean (SE)	5.18 (0.18)	4.45 (0.19)
Drinking days per week, mean (SE)	2.2 (0.05)	1.93 (0.05)
Average maximum drinks per occasion, mean (SE)	2.02 (0.08)	1.81 (0.06)
Total AUDIT score,^b^ mean (SE)	3.57 (0.16)	3.24 (0.11)

**Abbreviations: **AUDIT, Alcohol Use Disorders Identification Test; CAD, Canadian dollars; SD, standard deviation; SE, standard error. 

^a^ n = 1 missing. 

^b^ The AUDIT ranges from 0 (abstinence) to 40, with a score between 1 and 7 suggesting low-risk consumption of alcohol. 

***Weekly and combined guideline risk thresholds***

**Figure 1:**
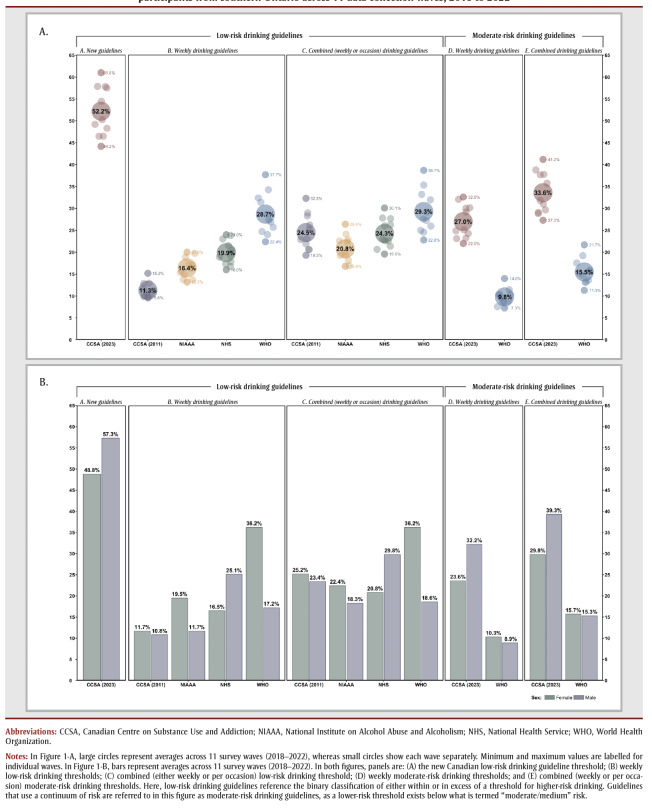
Percentage exceeding drinking guideline thresholds overall (A) and by sex assigned at birth (B) based on a sample of N = 1502 participants from southern Ontario across 11 data-collection waves, 2018 to 2022

Figure 1-A reveals the prevalence of exceeding the low- and moderate-risk drinking thresholds based on the Canadian and international benchmarks in aggregate (i.e. averaged over all time points) and over time. The specific aggregated differences in prevalence between Canada’s new low-risk drinking threshold and other guidelines are summarized in Table 2. (To illustrate differences in prevalence between Canada’s new low-risk drinking threshold and other guidelines in the year immediately prior to the introduction of the new Canadian DGs in 2023, a summary is provided in Supplemental Table 1, with demographics provided in Supplemental Table 2). On average, across survey waves, more than half (52.2%) of the sample were classified as exceeding the new low-risk drinking threshold of 2 drinks per week, 4.6 times those classified as exceeding the previous Canadian low-risk threshold (11.3%). In comparison to international weekly DGs, the proportion of those exceeding the new low-risk threshold was 3.2 times that of the NIAAA (16.4%); 2.6 times that of the NHS (19.9%); and 1.8 times the WHO (28.7%) low-risk thresholds. 

***Differences in prevalence by sex and age***

Table 3 summarizes the differences in prevalence exceeding new and previous Canadian low-risk drinking thresholds by sex and age. A higher percent of males (57.3%) exceeded the new low-risk drinking threshold than females (48.8%). This is in contrast to the previous Canadian low-risk drinking definition, which saw similar percentages of males and females exceeding the low-risk threshold (10.8% and 11.7%, respectively). This translates to a 5.3 times higher prevalence of males exceeding the new low-risk drinking threshold and a 4.2 times higher prevalence for females. Additional mean average prevalence of exceeding low- and moderate-risk Canadian and international DGs by sex can be found in Figure 1-B.

**Table 3:** Percentage exceeding new 2023 Canadian drinking guideline thresholds compared to previous 2011 Canadian guidelines overall, by sex assigned at birth and age, with prevalence multipliers, based on a sample of N = 1502 participants from southern Ontario across 11 data-collection waves, 2018 to 2022

Group	2023 Guidelines	2011 Guidelines	Mean (min–max) prevalence multipliers^a ^
% Exceeding low-risk guidelines	% Exceeding weekly guidelines	% Exceeding combined guidelines	a. Weekly	b. Combined
Overall
	52.2	11.3	24.5	4.6 (4.0–4.6)	2.1 (1.9–2.3)
Sex
Females	48.8	11.7	25.2	4.2 (3.7–4.2)	1.9 (1.7–2.1)
Males	57.3	10.8	23.4	5.3 (4.5–5.5)	2.4 (2.2–2.6)
Age (y)
<30	53.5	10.4	28.4	5.1 (4.6–6.1)	1.9 (1.7–2.2)
30–49	49.8	11.8	22.3	4.2 (3.2–4.8)	2.2 (2.2–2.4)
50+	52.5	12.3	19.6	4.3 (3.4–4.9)	2.7 (2.4–2.8)

**Abbreviation:** y, years. 

**Notes:** Percentage exceeding new 2023 and previous 2011 Canadian drinking guidelines (DGs), alongside prevalence multipliers at which the previous 2011 Canadian drinking guidelines would exceed classifying those by either the (a) weekly drinking threshold or (b) combined (i.e. either weekly or per occasion) drinking threshold using the new 2023 Canadian low-risk and moderate-risk drinking definitions. 

^a^ Prevalence multipliers can be interpreted as X number of times higher individuals in the sample would be classified as exceeding the new 2023 Canadian DGs relative to the previous 2011 Canadian DGs, and is calculated by dividing the average proportion of those exceeding the new 2023 drinking threshold by the average proportion of those exceeding the previous 2011 guidelines. 

The percent of young adults (<30 years of age) exceeding the new low-risk drinking threshold (53.5%) was similar to those aged 50 and older (52.5%), despite nearly a 9% difference between age categories using the previous combined low-risk drinking threshold (28.4% vs. 19.6%, respectively), which captures HED of young adults. The prevalence of those exceeding the low-risk drinking threshold among those under 30 years of age was 1.9 times higher compared to previous low-risk guidelines, while among those aged 50 and older it was 2.7 times higher.

***Awareness and perceptions of new guidelines***

**Figure 2:**
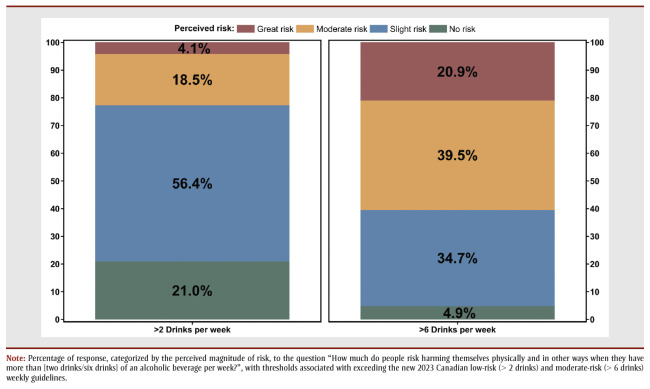
Perceived risk of exceeding 2023 Canadian low- and moderate-risk drinking guideline thresholds based on a sample of n = 1278 participants from southern Ontario, April 2023

Among the April 2023 subsample (n=1278), for which awareness and perceptions were assessed, 71.0% (n=908) reported alcohol consumption in the past month. Just over half of participants (51.1%) stated that they were aware of the new Canadian guidelines. This is lower than the 58.7% of Canadians surveyed in February 2023, although that survey only measured those who reported being aware of either the new or old Canadian guidelines.^26^ Furthermore, the majority (77.4%) perceived consumption of more than2 standard drinks per week to be of no or slight risk, compared to 22.6% who perceived this to be a moderate risk or greater (Figure 2). Exceeding the moderate threshold was generally seen as risky, with 60.4% of participants endorsing more than 6 standard drinks in a week as moderately risky or greater.

Table 4 presents the odds of perceiving drinking above the low-risk (>2 drinks) or moderate-risk (>6 drinks) thresholds as risky (moderate or higher risk). Females were 13% (odds ratio [OR] = 1.13, 95% confidence interval [CI]: 1.07–1.19; *p*<0.001) more likely to report more than 6 drinks a week as risky compared to males. There was no significant difference in the odds of females compared to males reporting greater than 2 drinks a week as risky (1.04, 0.99–1.09; *p*>0.05). Those aged 50 and older were 12% less likely to report drinking in excess of the low-risk threshold as risky (0.88, 0.83–0.93; *p*<0.001), and 14% less likely to report the moderate-risk threshold (0.86, 0.80–0.92; *p*<0.001) as risky compared to those under 30 years of age.

**Table 4:** Percentages and odds ratios of perceiving drinking in excess of the new 2023 Canadian low- and moderate-risk drinking guidelines as risky, by sex assigned at birth and age, in a sample of n = 1278 participants from southern Ontario, April 2023

	% Perceiving exceeding new guidelines as risky	Perceiving risk of > 2 drinks (exceeding low-risk thresholds)	Perceiving risk of > 6 drinks (exceeding moderate-risk thresholds)
>2 Drinks	>6 Drinks	OR (95% CI)^a ^	*p* value	OR (95% CI)^a ^	*p* value
Sex
Females	23.9	64.8	1.04 (0.99–1.09)	0.097	1.13 (1.07–1.19)	< 0.001
Males	20.5	53.4	1.00 (1.00–1.00)	—	1.00 (1.00–1.00)	—
Age (y)
<30	27.9	65.2	1.00 (1.00–1.00)	—	1.00 (1.00–1.00)	—
30–49	22.6	61.6	0.93 (0.88–0.98)	0.009	0.95 (0.89–1.01)	0.091
50+	15.5	52.2	0.88 (0.83–0.93)	< 0.001	0.86 (0.80–0.92)	< 0.001

**Abbreviations: **CI, confidence interval; OR, odds ratio; y, years. 

**Notes:** Percentage of n = 1278 participants perceiving drinking in excess of the new low- and moderate-risk drinking thresholds as risky (moderate or higher risk). Statistical significance of < 0.05 denoted in bold. 

^a ^Adjusted ORs and 95% CIs of main effects of sex assigned at birth and age, which control for reported alcohol consumption, awareness of the new guidelines, education level and yearly household income. 

## Discussion

This study examined the prevalence of individuals exceeding the new Canadian DGs compared to previous Canadian guidelines and other international benchmarks in a longitudinal sample of community adults. Comparison of prevalence revealed a greater magnitude of individuals exceeding the new low-risk drinking definition, with over half (52%) of the sample above the new Canadian low-risk threshold of no more than 2 drinks per week. This finding is in alignment with the estimate of 50% from a previous survey of a representative sample of Canadians from 2019.^27^ The prevalence of participants exceeding previous Canadian weekly guidelines of 11% is lower compared to the national Canadian estimate of 23%.^4^ However, using the national prevalence estimate would still imply a more than doubling of the proportion of individuals being classified as exceeding the new low-risk drinking threshold relative to the previous guidelines.

***Implications of universal guidelines across sex and age***

A higher percentage of males (57%) versus females (49%) exceeded the new low-risk drinking threshold, despite fewer males exceeding the previous low-risk threshold compared to females. This was a logical extension of the larger reduction in drinks for males proposed by the new guidance, combined with Canadian males typically consuming a greater volume of alcohol than females.^28^ This also follows similar findings using data from the 2019 CADS, which estimated that 62% of males versus 38% of females exceed the new weekly guidelines.^27^ Using the new low-risk drinking definition, which opted to omit prior sex-specific guidelines, males were found to exceed the guidelines 8.5% more than females; the previous guidelines only saw a 1% to 2% difference between the sexes. 

Although the move away from a sex binary is useful as it allows for simple messaging about alcohol-related dangers, it may also unintentionally imply that the absorption and metabolism of alcohol across sex is equal. The rationale for creating a single universal guideline across sexes is due to the risks being similar for females and males when consumption is within the new low-risk limits.^1^ However, with messaging focussing on a continuum of risk, emphasizing that lower consumption is safer, the differences in risk between the sexes and genders§.as consumption increases are unintentionally minimized. Specifically, these risks relate to: the differences in body size, body composition and pharmacokinetics, all of which can lead to greater sensitivity to alcohol in females;^29^ acute alcohol-related risks associated with sex and gender such as injury,^30^ sexual assault and intimate partner violence;^29^ and chronic risks such as a greater propensity for alcohol dependency in a shorter period of time (referred to as “telescoping”) for females.^31^ Therefore, stronger messaging on these sex- and gender-based risks would be beneficial.

Although there was a 9% difference in prevalence of those exceeding the low-risk threshold between young adults (<30) and adults aged 50 and older using the combined 2011 drinking threshold, that difference is reduced to 1% using the new low-risk threshold. As adults age, their typical pattern of consumption shifts from episodic drinking to more frequent but lower-quantity per episode drinking.^32^ This is pertinent because although previous guidelines might have defined frequent low-quantity drinking as low-risk (e.g. one drink/day), the new low-risk guidelines classify this pattern as exceeding both low- and moderate-risk thresholds. Notably, the lesser difference between young and older adult drinking patterns using the new guidelines demonstrates the potential for inherent differences in patterns of consumption to become muddled between these groups. From a public health perspective, the new DGs may be more pertinent for those aged 55 and older, given acute age-specific risks such as interactions with medication;^33^ accidents and falls;^34^ cognitive impairments;^35^ and other age-related physiological changes that reduce the ability to metabolize and protect against the negative effects of alcohol.^36^

Universal messaging on the harms of alcohol consumption emphasizes that any amount of alcohol consumption carries risk for all persons. Despite the inclusivity and simplicity of this message, the literature has highlighted that there should be a balance with specificity on the types of acute and chronic risks by sex/gender and age.^37^ As this study has exhibited, the prevalence of exceeding the new Canadian low-risk drinking threshold, unlike that of the previous guidelines, is not distributed evenly across either sex or age, so there may be a benefit for differential messaging in future public health efforts. Moreover, this study has also demonstrated that researchers should be cautious when leveraging the new low-risk drinking threshold; the high proportion of people exceeding the threshold alongside the potential to mask important differences in drinking patterns between subgroups may limit the utility of the new threshold in research contexts.

***Considerations for lower thresholds in research***

In addition to chronic risks related strictly to HED such as morbidity and mortality,^38^ HED also involves acute dose-dependent risks such as alcohol-attributed injuries resulting in emergency-room visits;^39^ suicide attempts;^40^ violence;^41^,^42^ and increases in alcohol-related problems.^43^ Thus, the use of both average weekly consumption and per occasion drinking thresholds provides a better estimate of risky drinking than just one metric alone.^9^,^32^

However, unlike other benchmarks, the new low-risk drinking definition utilizes the same 2-drink limit for both weekly and per occasion thresholds. The new guidelines emphasize that beyond 2 standard drinks, there is an increasing risk of acute harm coinciding with an increase in one’s blood alcohol concentration (BAC). Depending on biological factors and the timeframe in which the drinks are consumed, a BAC of 0.05% or higher** with just 3 standard drinks consumed across 2hours is possible for some.^44^ Although it is beneficial that the new guidelines highlight the existence of acute risks at the lower per occasion threshold, particularly as intoxicated individuals have a tendency to underestimate their level of intoxication,^45^ this also introduces more variability into the measure of acute risk prevalence, since body composition and timeframe of consumption may mean individuals retain a low BAC beyond 2 drinks. Therefore, researchers focussing on acute risks of HED may continue to leverage other established benchmarks such as the NIAAA binge drinking definition†† of 4+ and 5+ drinks for females and males, respectively, for which there is greater certainty that most individuals meeting this threshold would experience substantive psychoactive effects and be legally defined as intoxicated (with a BAC of 0.08%) when drinks are consumed over approximately 2 hours.^13^

Another potential research-related consideration of the new weekly and per occasion guidelines being quite different from previous Canadian or international guidelines is that comparisons to historical or international trends for population surveillance may prove more difficult. This is particularly true in research that may have only collected data on the percentage of people exceeding low-risk drinking definitions. Therefore, fine-grained alcohol use measures that can calculate various percentages of weekly and per occasion limits for use in future research studies would be most useful, such as the DDQ^20^ or Timeline Follow Back (TLFB^46^). This would allow for various thresholds of weekly averages and per occasion patterns (e.g. legal intoxication) to be calculated, ensuring future comparability of prevalence over time.

***Public awareness and perceptions***

Alcohol consumption is highly prevalent in Canada, so the substantial change in public health guidance present in the new guidelines may not resonate with many Canadians. Media coverage after the new DGs were released echoed this concern, with reports of hesitancy, and many people reporting that they do not plan on decreasing their alcohol consumption as a result of the new guidelines.^47^,^48^ Consistent with this, less than a quarter of the sample perceived there to be a moderate or higher risk in consuming more than 2alcoholic beverages per week. Although risk perception alone may not necessitate changes in behaviour,^49^ change is unlikely in the absence of a perception of alcohol-related harms. Anticipation of reduced drinking as a result of the new guidelines alone, therefore, absent extensive public awareness and education efforts, appears unlikely. Thus, if the goal is for these guidelines to have a national impact, additional strategies such as warning labels^50^,^51^ or DG promotional campaigns may be needed.

Other strategies that can help lower higher-risk drinking within a population are limitations on access to alcohol,^52^ restrictions on advertising^52^ and an increase in taxation.^52^,^53^ Indeed, these interventions are highlighted by the WHO’s SAFER initiative as cost-effective strategies to reduce the harm and burden of disease attributed to alcohol.^52^ Similarly, greater availability of alcohol due to the relaxation of legislation has been linked to increases in alcohol-related mortality,^54^ emergency-room visits^55^ and HED by young adults,^56^ all which have considerable health care and other costs to society.^57^,^58^

Across Canada, there is variability when it comes to restricting access to alcohol. In Ontario, the government has recently expanded access to alcohol by allowing the sale of alcohol in convenience and grocery stores, resulting in an estimated 8500 additional retail locations.^59^ Additionally, the Ontario government has halted an increase in taxes on alcohol since 2018 until at least 2026.^59^ The privatization of alcohol sales, which is expected to lower prices,^54^ may also result in an increase in alcohol consumption for Ontarians. If the goal of the new Canadian DGs is to lower population-level alcohol consumption to reduce alcohol-related harms, then provincial policies making alcohol easier to access and more affordable are in direct opposition of this goal, particularly as three-quarters of participants perceived more than 2 drinks per week as having little or no risk.

***Strengths and limitations***

These findings must be considered in the context of several strengths and limitations. First, the risk of temporal specificity of these findings has been reduced due to a large number of waves of data with high participant retention. Next, this study leveraged a relatively large longitudinal sample of nonclinical community adults that is fairly consistent with Canadian population demographics,^60^ albeit with more conservative rates of alcohol consumption and prevalence exceeding previous weekly guidelines than measured in the general population.^4^ However, despite similarities, the cohort is not a nationally representative sample, as evidenced by the lack of elevated rate of risky drinking among males that is present in population-based data,^4^,^61^ resulting in a lack of generalizability. In studies focussing on subgroups whose consumption of alcohol is much higher (e.g. youth, people with alcohol use disorder, etc.), the prevalence of those exceeding the low-risk drinking threshold will likely be even greater. 

The capturing of both typical frequency and drinking patterns among participants by the DDQ instrument was another strength of this study, representing an advantage over studies that typically use more succinct but less granular questions that ask about consumption over a specific threshold or ask participants to select their use pattern from a range of frequencies.^62^ Neither of these methods allows for a detailed examination of various drinking thresholds, nor do they allow for the combined limits to be examined. 

However, the DDQ instrument cannot capture those who consume alcohol intermittently, and thus more participants may surpass the per occasion drinking threshold, but on a less-than-weekly basis (e.g. such as every fortnight). Indeed, rates of underreporting due to imperfect measures have been quantified by researchers who found discrepancies between rates of drinking among the Canadian population and alcohol sales in Canada, estimating that over 50% of Canadians would exceed the moderate-risk weekly drinking threshold.^27^

§The CCSA’s technical report on the new guidelines highlights established risks of alcohol consumption by sex and gender, but these risks are not included in the more public-facing communications (e.g. summary infographic).**A BAC of 0.08% (the legal definition of intoxication) can also be possible. For example, using the NIAAA BAC calculator, the estimated BAC for a woman who weights 165 pounds and consumes 3 standard drinks over 2 hours is 0.08%.^44^††The technical CCSA report does make reference to the HED definition of 4+/5+ drinks for females/males, but it is not emphasized in any public-facing communication. This is logical, given that identifying an occasion limit that exceeded the weekly limit of 2 drinks would be counterintuitive to consumers.

## Conclusion

These findings indicate that in a sample of community adults over a four-year period (2018–2022), the new Canadian DGs more than quadruple the number of participants classified as exceeding low-risk thresholds compared to the previous guidelines, and increase the proportion relative to other international guidelines. The findings also reveal unequal risk of exceeding the new low- and moderate-risk drinking thresholds by sex, a result of omitting sex-specific guidelines and risks associated with patterns of use (e.g. HED). Findings also indicate that more than three-quarters of individuals perceived alcohol consumption in excess of the new 2-drink weekly limit as posing little to no risk. Those with a greater risk of exceeding the new DGs relative to previous guidelines are less likely to perceive consuming beyond drinking thresholds as risky, potentially exacerbating alcohol-related harms. Collectively, these results suggest that, if it is hoped that Canadians will adopt this guidance, major public education initiatives on the rationales for and importance of the new DGs will be necessary.

## Acknowledgements

The authors are grateful for the ongoing contribution of participants, as well as Jane De Jesus, Jessica Gillard, Laura Lee and Emily Vandehei. 

***Funding***

This study received funding from the Canadian Institutes of Health Research (Projects #420871, #437075, #487024), JM’s Peter Boris Chair in Addictions Research, JM’s Canada Research Chair in Translational Addiction Research (CRC-2020-00170) and a grant from the Michael G. DeGroote Centre for Medicinal Cannabis Research.

## Conflicts of interest

JM is a senior scientist and principal in BEAM Diagnostics, Inc., and has served as a consultant to Clairvoyant Therapeutics, Inc. There are no other conflicts of interest to declare.

## Authors’ contributions and statement

KB: conceptualization, methodology, investigation, data curation, formal analysis, writing—original draft, writing—review and editing. 

AD: conceptualization, methodology, investigation, writing—original draft, writing—review and editing. 

JM: conceptualization, methodology, investigation, writing—review and editing, funding acquisition, supervision. 

The content and views expressed in this article are those of the authors and do not necessarily reflect those of the Government of Canada.
